# Tailoring Quantum Tunneling in a Vanadium‐Doped WSe_2_/SnSe_2_ Heterostructure

**DOI:** 10.1002/advs.201902751

**Published:** 2019-11-27

**Authors:** Sidi Fan, Seok Joon Yun, Woo Jong Yu, Young Hee Lee

**Affiliations:** ^1^ Center for Integrated Nanostructure Physics (CINAP) Institute for Basic Science (IBS) Suwon 16419 Republic of Korea; ^2^ Department of Energy Science and Department of Physics Sungkyunkwan University Suwon 16419 Republic of Korea; ^3^ Department of Electrical and Computer Engineering Sungkyunkwan University Suwon 16419 Republic of Korea

**Keywords:** 2D tunneling heterojunctions, chemical vapor deposition, functional diodes, tin diselenide, vanadium‐doped tungsten diselenide

## Abstract

2D van der Waals layered heterostructures allow for a variety of energy band offsets, which help in developing valuable multifunctional devices. However, p–n diodes, which are typical and versatile, are still limited by the material choice due to the fixed band structures. Here, the vanadium dopant concentration is modulated in monolayer WSe_2_ via chemical vapor deposition to demonstrate tunable multifunctional quantum tunneling diodes by vertically stacking SnSe_2_ layers at room temperature. This is implemented by substituting tungsten atoms with vanadium atoms in WSe_2_ to provoke the p‐type doping effect in order to efficiently modulate the Fermi level. The precise control of the vanadium doping concentration is the key to achieving the desired quantum tunneling diode behaviors by tuning the proper band alignment for charge transfer across the heterostructure. By constructing a p–n diode for p‐type V‐doped WSe_2_ and heavily degenerate n‐type SnSe_2_, the type‐II band alignment at low V‐doping concentration is clearly shown, which evolves into the type‐III broken‐gap alignment at heavy V‐doping concentration to reveal a variety of diode behaviors such as forward diode, backward diode, negative differential resistance, and ohmic resistance.

## Graphical abstract

2D multifunctional diodes are realized by stacking CVD‐grown V‐doped WSe_2_ monolayers (p‐type) and SnSe_2_ (n‐type). Substituting W‐atoms with V‐atoms in WSe_2_ provokes the p‐type doping effect to modulate the Fermi level. The type‐II band alignment evolves into the type‐III broken‐gap alignment with increasing V‐doping concentration, revealing various diode behaviors such as forward, backward, and especially negative differential resistance transport.

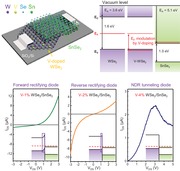

2D van der Waals (vdW) layered semiconductors possess various bandgaps ranging from 0.4 to 2.0 eV and different electron affinities, establishing predetermined heterojunctions of type‐I (straddling gap), type‐II (staggered gap), or type‐III (broken gap) band alignments.^1^, ^2^, ^3^, ^4^ Accordingly, a variety of functional devices can be developed by taking advantage of p–n junctions with different band alignments, which are the elementary building blocks for numerous applications in diodes, transistors, photodetectors, and solar cells.^5^, ^6^, ^7^, ^8^, ^9^ In addition, the charge transfer originating from the thermionic emission or quantum tunneling is affected by the band bending near the heterojunction, which relies on the Fermi level difference between materials.

Previously, carrier transport properties through a heterojunction appear to be in principle material‐dependent behaviors. Recent reports show the possibility to integrate multiple functions in a certain vdW heterostructure; for example, black phosphorus (BP) of different thicknesses are employed to provide a tunable Fermi level for the desired band bending.^10^, ^11^ Nevertheless, BP suffers from sensitivity in air.^12^ Besides, the use of mechanically exfoliated BP is not tenable for controlling the exact flake thickness, which directly determines its Fermi level, let alone realize accessible integration.

Chemical vapor deposition (CVD) technique is a promising approach in a wafer‐scale synthesis for industrial applications. Moreover, introducing the dopant during CVD growth is an effective way to change the carrier density in the material and thus modulate the position of the Fermi level.^13^ Here, we report a reliable and repeatable method to synthesize vanadium (V) substituted WSe_2_ monolayer by CVD and demonstrate multifunctional p–n diode behaviors in V‐doped WSe_2_/SnSe_2_ heterostructures. A liquid precursor with tungsten host and vanadium dopant atoms is adopted in our approach with only two zones for the precise control of V‐doping concentration in WSe_2_. The coverage of the grown‐WSe_2_ flakes is increased up to 90% at a high liquid precursor concentration (Figure S1, Supporting Information). In addition to the p‐type V‐doped WSe_2_ monolayer, multilayer SnSe_2_ as an n‐type material is introduced to construct the p–n junction. We observe the diverse p–n diode behaviors in V‐WSe_2_/SnSe_2_ devices with various V‐doping concentrations at room temperature, including quantum tunneling p–n diodes for the forward rectification, backward rectification, negative differential resistance (NDR), and ohmic resistance.

**Figure** **1**a,b is the schematics for synthesizing V‐doped WSe_2_ monolayer with the CVD approach. Liquid precursor containing solutions of ammonium metatungstate (AMT: W‐precursor) and ammonium metavanadate (AMV: V‐precursor) together with a promoter of alkali metal (NaOH) and iodixanol is spin‐casted on SiO_2_/Si substrate and then introduced into a two‐zone furnace CVD for the selenization process. The molar ratio of AMT to AMV in the liquid precursor is precisely controlled using a micropipette.

**Figure 1:**
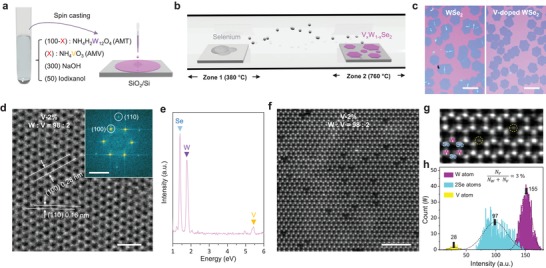
Synthesis and atomic structure of monolayer V‐doped WSe_2_. a) Schematic for preparation process of water‐based precursor. The precursor containing sodium metal (promoter), ammonium tungstate (W‐precursor), and ammonium vanadate (V‐precursor) are spin‐casted onto SiO_2_/Si wafer. b) Two‐zone furnace CVD process for growing V‐doped WSe_2_. The precursor‐coated substrate is introduced into the CVD chamber and is followed by selenization. c) Optical images of pure and V‐doped WSe_2_. The monolayer (0.8 nm) was retained up to V‐10% doping. The scale bar is 150 µm. d,e) High‐resolution TEM image (d) and EDS spectrum (e) of V‐2% WSe_2_. The scale bar is 1 nm. f,g) Unfiltered (f) and Gaussian‐filtered (g) ADF‐STEM images of V‐2% WSe_2_. After the filtering process, the substituted V atoms in W sites are clealy seen. The scale bar is 2 nm. h) The number of W, Se, and V atoms with the STEM intensity. The scale bar is 1 nm.

The hexagonal shape of flakes is preserved in V‐doped WSe_2_ samples (up to V‐10%), similar to the pristine WSe_2_ flakes (Figure 1c; Figure S2, Supporting Information). The V‐10% sample shows a uniform contrast in the entire flake area with a height profile of 0.8 nm, confirmed by atomic force microscope (AFM). The monolayer is maintained up to V‐10% doping (Figure S2, Supporting Information), at which multilayers are partially spotted. The highly crystalline atomic structure of V‐doped WSe_2_ monolayer (V‐2%) is observed without noticeable defect sites from the high‐resolution transmission electron microscopy (HRTEM) image with selective‐area electron‐diffraction pattern (Figure 1d). The interlayer distances of the (100) and (110) planes are identified to be 0.28 and 0.16 nm, respectively, in consistent with the reported values in WSe_2_.^14^

To explore V‐incorporation in WSe_2_, we further performed energy‐dispersive X‐ray (EDX) spectroscopy and annular dark field scanning TEM (ADF‐STEM). The chemical elements of V atom in addition to W and Se atoms were clearly discernible in EDX spectrum (Figure 1e) and EDX mapping (Figure S3, Supporting Information). Unlike the HRTEM image in Figure 1d, numerous dark spots are clearly distinguished in ADF‐STEM image (Figure 1f). The intensity of STEM is proportional to the atomic number (AN) by a*(AN)^*x*^, where *a* is constant and *x* ranges from 1.5 to 2.5.^15^ Therefore, the lowest atomic number of V (AN = 23) atoms are seen as the dark spots compared with brightest W (AN = 74) and Se (AN = 34) atoms. After Gaussian filtering (Figure 1g), V atoms are clearly seen by substituting W atoms. The average intensities of W (155), 2Se (97), and V (28) atoms are well fitted to a*(AN)^*x*^ (*a* = 0.29, *x* = 1.55), indicating that the dark spots are indeed V atoms (Figure 1h). Se‐vacancies are also presented in Figure S4 of the Supporting Information with a vacancy density of 1.7 × 10^13^ cm^−2^.

**Figure** **2**a demonstrates the Raman spectra of V‐doped WSe_2_ with different V‐doping concentrations (Figure S5, Supporting Information). The Raman shift near 250 cm^−1^ is ascribed to the mixed modes of E^1^_2g_ (strain) with A_1g_ (charge transfer) and is red‐shifted to 3 cm^−1^ at 4% V‐doping concentration. Such a red‐shift is dominantly ascribed to the A_1g_ mode by the p‐doping effect of V atoms on WSe_2_.^16^ The 2LA(M) mode appears around 260 cm^−1^ and is gradually developed as V‐doping concentration increases, reflecting the emergent crystalline disorders due to the randomly distributed V atoms.^17^ The photoluminescence (PL) is further conducted to study the p‐doping effect of V‐substitution to WSe_2_ (Figure 2b). The asymmetric PL emission near 1.66 eV is reminiscent of the positive trions in intrinsic p‐doped WSe_2_ (trion binding energy = ≈30 meV).^18^ As V‐doping concentration increases, the PL intensity is reduced due to charge (hole) screening and the position is red‐shifted by the developed positive trions.^19^

**Figure 2:**
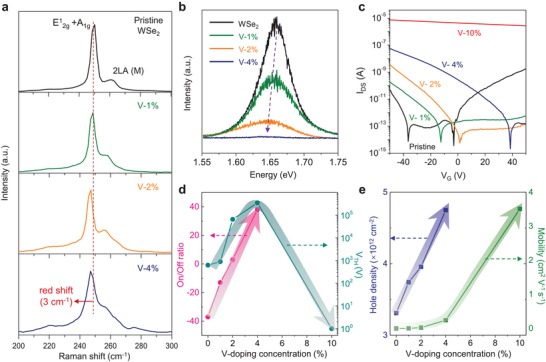
p‐Doping effect of V‐substitution to WSe_2_. a,b) Raman (a) and PL (b) spectra of V‐doped WSe_2_ monolayer at an excitation wavelength of 532 nm in terms of V‐doping concentration. c) *I*_DS_–*V*_G_ curves of V‐doped WSe_2_ transistors under *V*_DS_ = 0.5 V. d,e) On/off ratio (*I*_−50 V_/*I*_off_) and threshold voltage (d) field‐effect hole mobility and intrinsic hole carrier concentration (e) as a function of V‐doping concentration.

To investigate V‐doping effect on the electrical performance, we fabricated field effect transistors (FETs) using V‐doped WSe_2_ with Pd contact electrodes for efficient hole transport (Figure 2c). The pristine WSe_2_‐FET shows ambipolar transfer characteristics at a drain bias of 0.5 V. This prevails the p‐type behavior as V‐doping concentration increases. The threshold voltage in the hole region is shifted to the positive side, while the on/off current ratio reaches to ≈10^5^ at V‐4% doping (Figure 2d). At a high V‐doping concentration of 10%, the FET shows a heavily degenerate behavior with a negligible current modulation within the entire range of gate voltages (−50 to 50 V). Moreover, the hole mobility and carrier density increase as a function of V‐doping concentration (Figure 2e; for the detail calculation, see Note S1 and S2, and Figure S6, Supporting Information).

We next construct two‐terminal p–n diodes by vertically stacking the grown V‐doped WSe_2_ as a p‐type and the exfoliated SnSe_2_ as an n‐type, as shown schematically (**Figure** **3**a). The source and drain electrodes of Pd are deposited on the V‐WSe_2_/SnSe_2_ heterostructure (Figure 3b; for the detail fabrication process, see Figure S7, Supporting Information).^20^, ^21^ The thickness of SnSe_2_ flakes in all diodes were 11–17 nm, as confirmed by AFM for the controlled experiments (Figure S8, Supporting Information). The Fermi level of the V‐doped WSe_2_ is modulated with V‐doping concentration. Meanwhile, the multilayer SnSe_2_ FET device reveals a heavily degenerate n‐type behavior by the high n*‐t*ype current of ≈100 µA µm^−1^ (Figure S8, Supporting Information). As V‐doping concentration increases, the Fermi level is shifted toward the valence band edge up to V‐4% and further into the valence band at V‐10%, implying a near broken‐gap band alignment with n‐type SnSe_2_ (Figure 3c).^22^

**Figure 3:**
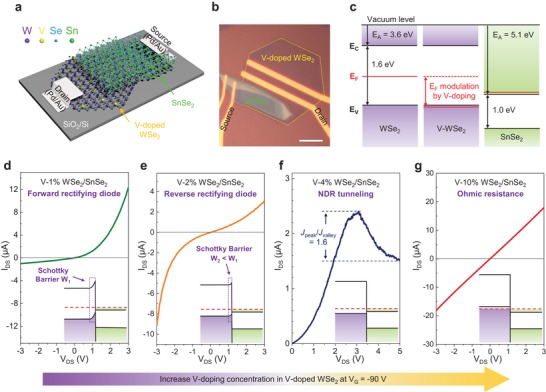
Device structure and electrical performance of two‐terminal diodes from CVD‐synthesized V‐doped WSe_2_ and exfoliated SnSe_2_. a) Schematic showing p‐type V‐WSe_2_ at the bottom and n‐type SnSe_2_ on top, with both Pd contacts. b) Optical image of a representative device of V‐2%. The scale bar is 10 µm. c) Band structures of V‐WSe_2_ and SnSe_2_ with their electron affinities and bandgaps, suggesting near broken‐gap alignment in the heterostructures. The Fermi levels are estimated from the threshold voltages. d–g) *I*_DS_–*V*_DS_ characteristics (linear scale at room temperature) with diverse diode behaviors corresponding to different V‐doping concentrations in WSe_2_. The inset figures are the band alignments simulated by Poisson's equation when SnSe_2_ was brought into contact with V‐doped WSe_2_. All the devices are gated at −90 V.

We now demonstrate a variety of diode behaviors in V‐WSe_2_/SnSe_2_ devices by varying the V‐doping concentration at room temperature. The diode device at V‐1% (Figure 3d) as well as the pristine WSe_2_ device (Figure S9, Supporting Information) shows a typical forward rectifying diode behavior. The Fermi level of V‐1% or pristine WSe_2_ sample locates near the middle of the bandgap, allowing for a large current flow from WSe_2_ to SnSe_2_ driven by the forward bias while suppressing the reverse current. At V‐2% doping, the Fermi level downshifts closer to the valence band. An asymmetric ambipolar transistor is manifested, preferring a backward rectifying diode behavior (Figure 3e). A more prominent device is constructed at V‐4% doping. NDR behavior is clearly observed in the forward bias region from 2.8 to 5.0 V with a peak‐to‐valley ratio of 1.6 (Figure 3f). When the Fermi level of n‐SnSe_2_ is located above the valence band edge of V‐doped WSe_2_, the current drops gradually with the reduction in electron band‐to‐band tunneling (BTBT). At a high V‐doping concentration (V‐10%), the V‐doped WSe_2_ turns into the degenerate state with the Fermi level getting inside the valence band, which is confirmed by the subtle gating effect in the entire range of gate bias (Figure S10, Supporting Information). In such a case, the diode behaves as an ohmic resistance due to the presence of both degenerate n‐SnSe_2_ and degenerate p‐WSe_2_ (Figure 3g). The contact issues of resistance are excluded here owing to the near‐ohmic contact in V‐doped WSe_2_/Pd junctions (Figure S11, Supporting Information).^10^

The band bending of alignments in heterostructures occurs in planar V‐doped WSe_2_ rather than the vertical V‐WSe_2_/SnSe_2_ junction due to the thin thickness of monolayer V‐WSe_2_ 9, 23 and low carrier density while the V‐doping concentration is low. In view of the SnSe_2_, which is degenerate and appears in metal, the band bending can be negligible. According to Poisson's equation, band alignments in the V‐WSe_2_/SnSe_2_ heterojunctions are calculated upon different V‐doping concentrations in V‐doped WSe_2_ (insets in Figure 3d–g; the details are provided in Note S3 and Figure S12, Supporting Information). The band bending (depletion region width) in the V‐WSe_2_ at low V‐doping concentration are much longer than the atomic thickness of monolayer V‐WSe_2_, inducing the bands bend along the planar direction in V‐WSe_2_. The initial type II band alignment is preserved at low V‐concentration (up to V‐4%). Further increase V‐concentration to V‐10%, the width of the depletion layer decreases, eventually obtains a type III broken‐gap alignment, implying the evolution of the carrier transport mechanism.

The aforementioned appealing device characteristics modulated by V‐doping concentration are further improved for high device efficiency via electrostatic doping. The *I*_DS_–*V*_DS_ output curves are measured at various gate biases from −30 to −110 V (**Figure** **4**). In addition to the p‐type V‐doping in WSe_2_, the hole doping by the negative gate bias is applied to boost the efficiency without altering the device characteristics.^24^, ^25^ The Fermi level in heavily n‐doped SnSe_2_ is strongly pinned. In the forward rectifying diode at 1% V‐WSe_2_ (Figure 4a), a high current is achieved at a large negative gate bias; however, it yields a high rectification ratio (*I*_3V_/*I*_−3 V_) of 135 at *V*_G_ = −30 V, benefiting from the suppression of minority carrier drift in the off‐state compared to that at *V*_G_ = −110 V. For the backward rectifying diode, the rectification ratio (*I*_−3 V_/*I*_3V_) increases up to 2.8 at *V*_G_ = −110 V (Figure 4b). Meanwhile, the peak‐to‐valley ratio in NDR diode rises up to 2.3 at *V*_G_ = −110 V (Figure 4c). Similar device characteristics are obtained from other four devices with the corresponding V‐doping concentration (Figure S13, Supporting Information). Figure 4d summarizes the improved device performances in terms of the rectification ratio (forward and backward) and peak‐to‐valley ratio with the gate bias modulation. The reproducibility of our devices is ensured with reasonable error bars. As the negative gate bias increases, the rectification ratio degrades in the forward direction but is improved in the backward diode. The peak‐to‐valley ratio is inversely proportional to the gate bias with a maximum value of 2.3, especially with 4% V‐WSe_2_, higher than the value of 1.8 obtained from the WSe_2_/SnSe_2_ without h‐BN insulating layer.^26^ This is ascribed to the increase in available energy states for tunneling, modulated by the high V‐doping concentration.

**Figure 4:**
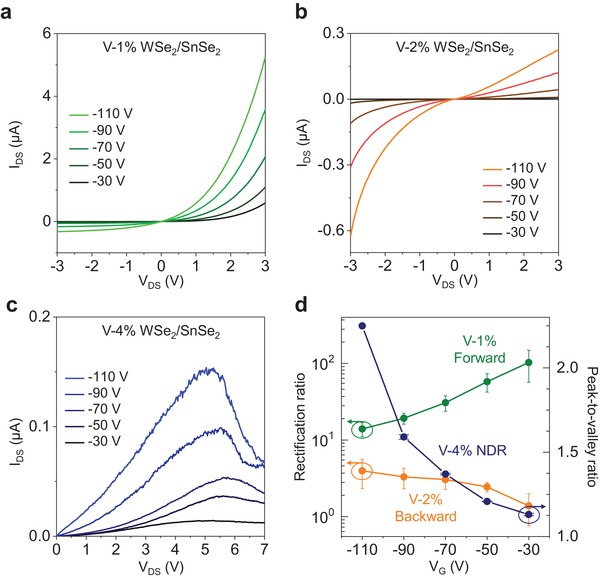
Gate‐tunable behaviors of V‐doped WSe_2_/SnSe_2_ devices. a–c) *I*_DS_–*V*_DS_ output curves of the forward rectifying diode, backward rectifying diode, and NDR tunneling under various gate biases. d) Device rectification ratio (*I*_3V_/*I*_−3 V_ in the forward direction, and *I*_−3 V_/*I*_3V_ in the backward direction) and NDR peak‐to‐valley ratio as a function of gate bias with reasonable error bars, demonstrating the reproducibility.

Diverse diode behaviors indicate different carrier transport mechanisms. In the pristine WSe_2_ or V‐1% device, there is a depletion region in WSe_2_ nearby the heterointerfaces (Figure S12a, Supporting Information). Under a positive drain bias (**Figure** **5**a), the energy bands of WSe_2_ shift down, thinning the barrier width. The majority electrons residing in the SnSe_2_ conduction band, therefore, overcome the triangular barrier and migrate to the other side, generating a forward current. This process is dominated by the thermionic Fowler–Nordheim (FN) tunneling or FN tunneling, which can be modeled as^27^

(1) $$I\left(V\right)\;=\frac{Aq^{3}mV^{2}}{8\pi h\varphi W^{2}m^{*}}\;\exp\left[\frac{-8\pi \sqrt{2m^{*}}\varphi^{3/2}W}{3hqV}\;\right]$$

where *A* is the effective contact area, *q* is the unit charge, *h* is the Planck's constant, *m* and m* are the electron free mass and effective mass, respectively, and *W* and φ are the barrier width and height, respectively. Based on the derivation, 1/*V* follows ln(*I*/*V* ^2^) in the relationship of negative linear correlation. The ln(*I*/*V* ^2^)‐1/*V* plot (Figure S14, Supporting Information) is calculated from Figure 3d, confirming FN tunneling mechanism with the negative slop at a high drain bias (near zero).^28^ When *V*_DS_ < 0, the minority carrier drift starts to dominate the current flowing from SnSe_2_ to WSe_2_. Since the intrinsic concentration of minority carriers is limited and the diffusion length stays identical, the reverse current is small and tends to saturate, showing a forward rectifying behavior.

**Figure 5:**
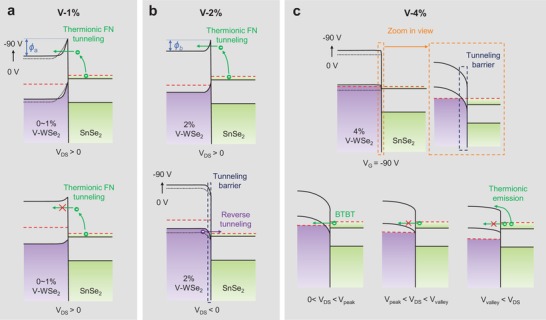
Band alignments in various V‐doped WSe_2_/SnSe_2_ devices. a) In the pristine WSe_2_ and V‐1% devices, driven by a positive drain bias, the majority electrons thermally excited from the SnSe_2_ conduction band dominate the carrier transport via FN tunneling, generating a forward rectifying current. ϕ_a_ is the built‐in potential. b) In the V‐2% device, when *V*_DS_ < 0, electrons tunnel from the WSe_2_ valence band into the empty conduction band of SnSe_2_ by the band‐to‐band tunneling, exhibiting a backward rectifying behavior. ϕ_b_ is the built‐in potential. c) In the V‐4% WSe_2_, the Fermi level is expected to enter its valence band at a high negative gate due to the strong accumulation of holes. A tunneling channel is thereby established in the device for the band‐to‐band tunneling. Electrons are transferred across the tunneling barrier at the interface with an NDR behavior in the positive drain bias region.

The Fermi level in V‐2% WSe_2_ locates closer to its valence band edge compared to that in pristine or V‐1% sample because of the V‐doping effect, and further approaches at a high negative gate bias. When *V*_DS_ > 0, the SnSe_2_ conduction band aligns with the forbidden band of WSe_2_, resulting in the forward FN tunneling current as in pristine WSe_2_ or V‐1% device (upper panel in Figure 5b). The depletion region in V‐2% WSe_2_ is much thinner but the built‐in potential shows ϕ_b_ < ϕ_a_ (marked in upper panels in Figure 5b and Figure 5a, respectively) as indicated in the calculated band alignment (Figure S12b, Supporting Information), leading to a smaller forward current. Meanwhile, the energy bands of V‐WSe_2_ move upward when *V*_DS_ < 0, which allows for electrons from the WSe_2_ valence band heading to the empty states in the SnSe_2_ conduction band, generating a backward BTBT current. In this case, the thinner tunneling barrier promotes the efficient band‐to‐band tunneling in backward, whereas the lower built‐in potential reduces the forward thermionic current, forming the backward rectifying behavior.

As V‐4% WSe_2_‐FET shows a strong p‐type property, the original Fermi level is expected to locate near its valence band edge (Figure S12c, Supporting Information). Gated by a high negative bias (Figure 5c), the electrostatic doping pushes the Fermi level into the valence band due to the strong accumulation of holes, leaving large amounts of empty states. Driven by a small positive drain bias (0 < *V*_DS_ < *V*_peak_), electrons from the SnSe_2_ conduction band tunnel into the WSe_2_ valence band owing to the overlap of equivalent energy states, and thus the forward tunneling current is generated. This current then reaches its peak when the SnSe_2_ Fermi level matches the WSe_2_ valence band edge, where the populations of equivalent states in two sides reach a maximum. A further increase in *V*_DS_ induces the reduction in the tunneling current, namely, NDR behavior. The upper part of the SnSe_2_ conduction band aligns with the forbidden band of WSe_2_, thereby prohibiting the BTBT of electrons, and the overlap region shrinks continuously until vanishes. Normally, *I*_DS_ would pick up after the valley point and be dominated by the thermionic emission. However, this part is absent in some of our devices, which is probably because of the tunneling barrier or large hysteresis. Most of the drain bias drops at the wide interface barrier, which results in the appearance of peak current at a high *V*_DS_. In addition, the large hysteresis in the V‐4% WSe_2_ transistor (Figure S15a, Supporting Information) indicates the carrier trapping or scattering, which severely impedes the majority carrier diffusion. Apart from this, device with apparent *I*_DS_ pick‐up behavior is provided in Figure S15b of the Supporting Information with a peak‐to‐valley ratio of 1.4.

In conclusion, we have shown the tunable quantum tunneling in the V‐doped WSe_2_/SnSe_2_ heterostructure by varying the doping concentration at room temperature. A liquid precursor containing transition metal and vanadium dopant atoms is used in our approach to synthesize V‐WSe_2_ via CVD. The precise control of the doping concentration efficiently modulates the Fermi level position, achieving the p‐type doping effect. This method induces the substitution of W by V, endowing a proper band bending for the desired band alignment, which determines the charge transfer across the heterostructure. We theoretically probed the variation in the band alignment upon to different V‐doping concentration, where the intrinsic type‐II band alignment is preserved at low concentration, and evolves into the type‐III broken‐gap alignment by heavily doping. Hence, the realization of diverse functions with the forward rectifying diode, backward rectifying diode, NDR diode, and ohmic resistance has been illustrated. They therefore suggest the promising opportunities to be applied for future integration in the scaled electronics.

## Experimental Section

*Preparation of Liquid Precursor*: The precursor solution was prepared by mixing four types of water‐based solutions: (i) tungsten precursor (ammonium metatungstate: NH_4_H_2_W_12_O_4_), (ii) vanadium precursor (ammonium metavanadate: NH_4_VO_3_), (iii) promoter (sodium hydroxide: NaOH), and (iv) medium solution (iodixanol). The V‐doping concentration in V‐doped WSe_2_ was adjusted by mixing the molar ratio of tungsten to vanadium precursor. The mixed solution was then coated onto SiO_2_/Si wafer by spin‐casting at 3000 rpm for 1 min.

*Synthesis of V‐Doped WSe_2_ by CVD*: An atmospheric CVD system with two‐zone furnaces was used for separately controlling the temperature of the selenium source and reaction zone. Here, 0.5 g of selenium (Sigma, 209643) was introduced, while the precursor‐coated substrate was placed in the reaction zone. For the growth of V‐doped WSe_2_, the selenium zone was heated up to 400 °C at a rate of 50 °C per min. At the same time, the substrate zone was set to 760 °C. Nitrogen gas of 500 sccm and hydrogen gas of 25 sccm were injected during CVD.

*TEM Measurement*: TEM and ADF‐STEM images were taken by a probe aberration‐corrected JEM ARM 200F machine, operated at 80 kV for high‐resolution TEM observations. The imaging time was set within 1 min under a high‐magnification STEM node to avoid beam damage on the monolayer V‐doped WSe_2_ samples.

*Device Fabrication*: The fabrication of the V‐WSe_2_/SnSe_2_ devices consists of three steps: (i) pristine WSe_2_ or V‐WSe_2_ flakes transfer, (ii) exfoliation of SnSe_2_ flakes and dry transfer in the glovebox, and (iii) electrode metallization. First, the CVD grown WSe_2_ flakes were transferred onto a 300 nm SiO_2_/Si substrate by wet‐etching using hydrofluoric acid. Second, the SnSe_2_ flakes were exfoliated using a Scotch tape onto a poly propylene carbonate (PPC)/300 nm SiO_2_/Si substrate, visualizing the thin and large‐size flakes. The desired SnSe_2_ flake along with the PPC film was then mechanically peeled off from the silicon substrate and attached onto a polydimethylsiloxane stamp. Next, SnSe_2_ and WSe_2_ were brought together, followed by poly methyl methacrylate spin‐coating prior to performing electrode metallization for protection. All the manipulations of SnSe_2_ flakes were performed in the glovebox. Finally, the source (S) and drain (D) contacts (Pd/Au of 5/50 nm) for both WSe_2_ and SnSe_2_ were patterned by e‐beam lithography prior to metal deposition. The entire fabrication process was schematically illustrated in Figure S7 of the Supporting Information.

*Device Characterization*: Electrical measurements were conducted by a probe station equipped with source/measurement units (Keithley 4200 and Agilent B2900A) at the high vacuum (10^−6^ Torr). AFM mapping analyses were recorded in a SPA400 (SEIKO) system. NT‐MDT Raman & PL spectroscopy (532 nm laser) were used to study the samples.

## Conflict of Interest

The authors declare no conflict of interest.

## Supporting information

Supporting Information

## References

[1] J. Shim , S. Oh , D. Kang , S. Jo , M. H. Ali , W. Choi , K. Heo , J. Jeon , S. Lee , M. Kim , Y. J. Song , J. Park , Nat. Commun. 2016, 7, 13413. 27819264 10.1038/ncomms13413 PMC5103069

[2] X. Hong , J. Kim , S. Shi , Y. Zhang , C. Jin , Y. Sun , S. Tongay , J. Wu , Y. Zhang , F. Wang , Nat. Nanotechnol. 2014, 9, 682. 25150718 10.1038/nnano.2014.167

[3] M. Li , M. Z. Bellus , J. Dai , L. Ma , X. Li , H. Zhao , X. C. Zeng , Nanotechnology 2018, 29, 335203. 29790862 10.1088/1361-6528/aac73a

[4] H. Fang , C. Battaglia , C. Carraroc , S. Nemsak , B. Ozdol , J. S. Kang , H. A. Bechtel , S. B. Desai , F. Kronast , A. A. Unal , G. Conti , C. Conlon , G. K. Palsson , M. C. Martin , A. M. Minor , C. S. Fadley , E. Yablonovitch , R. Maboudian , A. Javey , Proc. Natl. Acad. Sci. USA 2014, 111, 6198. 24733906 10.1073/pnas.1405435111 PMC4035947

[5] R. Cheng , D. Li , H. Zhou , C. Wang , A. Yin , S. Jiang , Y. Liu , Y. Chen , Y. Huang , X. Duan , Nano Lett. 2014, 14, 5590. 25157588 10.1021/nl502075n PMC4189621

[6] L. Britnell , R. M. Ribeiro , A. Eckmann , R. Jalil , B. D. Belle , A. Mishchenko , Y. Kim , R. V Gorbachev , T. Georgiou , S. V Morozov , A. N. Grigorenko , A. K. Geim , C. Casiraghi , A. H. C. Neto , K. S. Novoselov , Science 2013, 340, 1311. 23641062 10.1126/science.1235547

[7] W. J. Yu , Y. Liu , H. Zhou , A. Yin , Z. Li , Y. Huang , X. Duan , Nat. Nanotechnol. 2013, 8, 952. 24162001 10.1038/nnano.2013.219 PMC4249654

[8] W. J. Yu , Z. Li , H. Zhou , Y. Chen , Y. Wang , Y. Huang , X. Duan , Nat. Mater. 2013, 12, 246. 23241535 10.1038/nmat3518 PMC4249642

[9] C. Lee , G. Lee , A. M. Van Der Zande , W. Chen , Y. Li , M. Han , X. Cui , G. Arefe , C. Nuckolls , T. F. Heinz , J. Guo , J. Hone , P. Kim , Nat. Nanotechnol. 2014, 9, 676. 25108809 10.1038/nnano.2014.150

[10] X. Liu , D. Qu , H. Li , I. Moon , F. Ahmed , C. Kim , M. Lee , Y. Choi , J. H. Cho , J. C. Hone , W. J. Yoo , ACS Nano 2017, 11, 9143. 28787570 10.1021/acsnano.7b03994

[11] P. K. Srivastava , Y. Hassan , Y. Gebredingle , J. Jung , B. Kang , W. J. Yoo , B. Singh , C. Lee , Small 2019, 15, 1804885. 10.1002/smll.201804885 30730094

[12] Y. Wang , B. Yang , B. Wan , X. Xi , Z. Zeng , E. Liu , G. Wu , Z. Liu , W. Wang , 2D Mater. 2016, 3, 035025.

[13] S. J. Yun , G. H. Han , H. Kim , D. L. Duong , B. G. Shin , J. Zhao , Q. A. Vu , J. Lee , S. M. Lee , Y. H. Lee , Nat. Commun. 2017, 8, 2163. 29255139 10.1038/s41467-017-02238-0 PMC5735184

[14] S. J. Yun , S. M. Kim , K. K. Kim , Y. H. Lee , Curr. Appl. Phys. 2016, 16, 1216.

[15] S. Yamashita , J. Kikkawa , K. Yanagisawa , T. Nagai , K. Ishizuka , K. Kimoto , Sci. Rep. 2018, 8, 12325. 30120323 10.1038/s41598-018-30941-5 PMC6098135

[16] C.‐H. Chen , C.‐L. Wu , J. Pu , M. H. Chiu , P. Kumar , T. Takenobu , L.‐J. Li , 2D Mater. 2014, 1, 034001.

[17] S. Mignuzzi , A. J. Pollard , N. Bonini , B. Brennan , I. S. Gilmore , M. A. Pimenta , D. Richards , D. Roy , Phys. Rev. B 2015, 91, 195411.

[18] G. Wang , L. Bouet , D. Lagarde , M. Vidal , A. Balocchi , T. Amand , X. Marie , B. Urbaszek , Phys. Rev. B 2014, 90, 075413. 10.1103/PhysRevLett.114.097403 25793850

[19] W. T. Kang , I. M. Lee , S. J. Yun , Y. Il Song , K. Kim , D. H. Kim , Y. S. Shin , K. Lee , J. Heo , Y. M. Kim , Y. H. Lee , W. J. Yu , Nanoscale 2018, 10, 11397. 29877543 10.1039/c8nr03427c

[20] Y. Wang , R. X. Yang , R. Quhe , H. Zhong , L. Cong , M. Ye , Z. Ni , Z. Song , J. Yang , J. Shi , J. Li , J. Lu , Nanoscale 2016, 8, 1179. 26666570 10.1039/c5nr06204g

[21] H. Fang , S. Chuang , T. C. Chang , K. Takei , T. Takahashi , A. Javey , Nano Lett. 2012, 12, 3788. 22697053 10.1021/nl301702r

[22] C. Zhang , C. Gong , Y. Nie , K. Min , C. Liang , Y. J. Oh , H. Zhang , W. Wang , S. Hong , L. Colombo , R. M. Wallace , K. Cho , 2D Mater. 2017, 4, 015026.

[23] A. Nourbakhsh , A. Zubair , M. S. Dresselhaus , T. Palacios , Nano Lett. 2016, 16, 1359. 26784325 10.1021/acs.nanolett.5b04791

[24] X. Zhou , X. Hu , S. Zhou , H. Song , Q. Zhang , L. Pi , L. Li , H. Li , J. Lü , T. Zhai , Adv. Mater. 2018, 30, 17032868. 10.1002/adma.201703286 29315847

[25] T. Roy , M. Tosun , M. Hettick , G. H. Ahn , C. Hu , Appl. Phys. Lett. 2016, 108, 083111.

[26] S. Fan , Q. A. Vu , S. Lee , T. L. Phan , G. Han , Y. Kim , W. J. Yu , Y. H. Lee , ACS Nano 2019, 13, 8193. 31260265 10.1021/acsnano.9b03342

[27] R. H. Fowler , L. Nordheim , Proc. R. Soc. A 1928, 119, 173.

[28] Q. A. Vu , J. H. Lee , V. L. Nguyen , Y. S. Shin , S. C. Lim , K. Lee , J. Heo , S. Park , K. Kim , Y. H. Lee , W. J. Yu , Nano Lett. 2017, 17, 453. 27983857 10.1021/acs.nanolett.6b04449

