# Supplementary material for: Tailoring Quantum Tunneling in a Vanadium‐Doped WSe2/SnSe2 Heterostructure
Source: Adv Sci (Weinh). 2019 Nov 27;7(3):1902751. doi: 10.1002/advs.201902751 (PMC7001641; doi:10.1002/advs.201902751)
Supplement: Supplementary file 1 — Supporting Information [file ADVS-7-1902751-s001.pdf]

Copyright WILEY-VCH Verlag GmbH & Co. KGaA, 69469 Weinheim, Germany, 2019.

# Supporting Information

for *Adv. Sci.,* DOI: 10.1002/advs.201902751

# Tailoring Quantum Tunneling in a Vanadium-Doped $WSe_2/SnSe_2$ Heterostructure

*Sidi Fan, Seok Joon Yun, Woo Jong Yu, and Young Hee Lee**

Copyright WILEY-VCH Verlag GmbH & Co. KGaA, 69469 Weinheim, Germany, 2019

# Supporting Information

**Tailoring Quantum Tunneling in Vanadium-doped $WSe_2$/$SnSe_2$ Heterostructures**

*Sidi Fan, Seok Joon Yun, Woo Jong Yu, and Young Hee Lee*[*]

[*]Correspondence to: leeyoung@skku.edu

**Note1. Field-effect hole mobility calculation**

The field-effect hole mobility $\mu_h$ is obtained from the following equations,

$$\mu_h = \frac{L g_m}{W C_i V_{DS}} \qquad (1)$$

where $L$ and $W$ are the length and width of the carrier transport channel, respectively, $g_m$ is the transconductance, $C_i$ is the capacitance of the gate dielectric layer, and $V_{DS}$ is the source-drain bias.

$$g_m = \frac{dI_{DS}}{dV_G} \qquad (2)$$

where $I_{DS}$ is the source-drain current at the gate bias of $V_G$.

$$C_i = \frac{\varepsilon_0 \varepsilon_i}{d_i} \qquad (3)$$

where $\varepsilon_0$ is the vacuum permittivity ($8.854\times10^{-12}$ $Fm^{-1}$), $\varepsilon_i$ is the relative permittivity of $SiO_2$, and $d_i$ is the thickness of $SiO_2$.

In our devices, $L/W$ is equal to 0.18, 0.31, 0.14, 0.27, and 0.17 in pristine $WSe_2$, 1%, 2%, 4%, and 10% V-$WSe_2$ FETs, respectively. $V_{DS}$ = 0.5 V, $\varepsilon_i$ = 3.9, $d_i$ = 300 nm, and $g_m$ at a given gate bias is extracted from the transfer curve in Figure 2c. The field-effect hole mobilities in each device are shown in Figure S5. The highest values of each curve are taken for comparation in Figure 2e.

**Note2. Hole carrier concentration calculation**

The hole carrier concentration is calculated from $p = q^{-1} C_i |V_{th} - V_G|$, where $q$ is the unit charge, $V_{th}$ is the threshold voltage, and $V_G$ is the gate bias. In the pristine $WSe_2$ transistor, $V_{th\text{-}0}$ = -46 V (from the linear scale). Thus, $p_0 = 3.31 \times 10^{12}$ cm$^{-2}$ at $V_G$ = 0 V. Due to the $p$-doping effect of V-substitution in $WSe_2$, the hole carrier concentration in V-$WSe_2$ is added to that in pristine $WSe_2$. Therefore, $p_{dope} = p_0 + q^{-1} C_i |V_{th\text{-}dope} - V_{th\text{-}0}|$.

**Note3. Band alignment simulations with various V-doping concentrations**

As $SnSe_2$ is an intrinsically degenerate material and V-$WSe_2$ is a monolayer, the band bending in the heterostructure would take place in the planar direction instead of the vertical direction.[1] The configurations of V-$WSe_2$ (right) and $SnSe_2$ (left) in the planar direction (Figure S11) are simulated based on Poisson's equation. We use a model similar as the previous one to calculate the charge distribution and obtain the band alignment in the heterostructures.[2],[3] Because of the semiconductor property of both V-$WSe_2$ and $SnSe_2$, depletion approximation with uniform charge carrier is applied, where the charge density is equal to the doping level in the materials. The electron doping concentration in $SnSe_2$ is reported to be $N_d$ = ~$10^{19}$ cm$^{-3}$.[4] The hole carrier density in V-$WSe_2$ is gradually increased along the V-concentration as shown in Figure 2c. In our simulation, $N_a$ is chosen to be $10^{14}$, $10^{17}$, $10^{19}$, and $10^{23}$ cm$^{-3}$ to estimate the band alignment as a function of V-concentration. The Fermi level in the semiconductor is

$$W_{f_n} = E_c + \frac{E_g}{2} - \frac{K_b T}{q} \ln \frac{N_d}{n_i} \qquad (4)$$

for $SnSe_2$, and

$$W_{fp} = E_c + \frac{E_g}{2} + \frac{K_b T}{q} \ln \frac{N_a}{n_i} \qquad (5)$$

for $WSe_2$, where $E_c$ is the conduction band, $E_g$ is the band gap, $K_b$ is the Boltzmann constant, $T$ is the temperature, $q$ is the unit charge, and $n_i$ is the intrinsic doping level, which is set to be $10^{10}$ cm$^{-3}$ in the simulation. Thus, the barrier height in V-$WSe_2$/$SnSe_2$ is

$$V_{bi} = W_{fn} - W_{f_p} + V_{DS} \qquad (6)$$

under the applied source-drain bias of $V_{DS}$. The depletion layer width is estimated to be

$$x_n = \sqrt{\frac{2\varepsilon_n V_{bi}}{q}\frac{N_a}{N_d(N_a+N_d)}} \qquad (7)$$

in $SnSe_2$, and

$$x_p = \sqrt{\frac{2\varepsilon_p V_{bi}}{q}\frac{N_d}{N_a(N_a+N_d)}} \qquad (8)$$

in $WSe_2$, where $\varepsilon_n$ and $\varepsilon_p$ are the dielectric constants of $SnSe_2$ and $WSe_2$, respectively. Based on Poisson's equation, the voltage potential of $V(x)$ at the $x$ position in the planar direction is

$$V(x) = \frac{qN_d}{2\varepsilon_n}(x + x_n)^2,\ -x_n \le x \le 0 \qquad (9)$$

$$V(x) = \frac{qN_a}{\varepsilon_p}\left(x_p x - \frac{x^2}{2}\right) + \frac{qN_d}{2\varepsilon_n}{x_n}^2,\ 0 \le x \le x_p \qquad (10)$$

$$V(x) = \frac{qN_a}{\varepsilon_p}\left({x_p}^2 - \frac{{x_p}^2}{2}\right) + \frac{qN_d}{2\varepsilon_n}{x_n}^2,\ x_p \le x \qquad (11)$$

The above equations are solved self-consistently to obtain the band diagrams. As shown in Figure S11, an initial type II band alignment is formed by the low V-concentration. With the increase in $N_a$, the depletion layer width is gradually decreased, and eventually a type III broken gap alignment is formed by the high V-concentration.

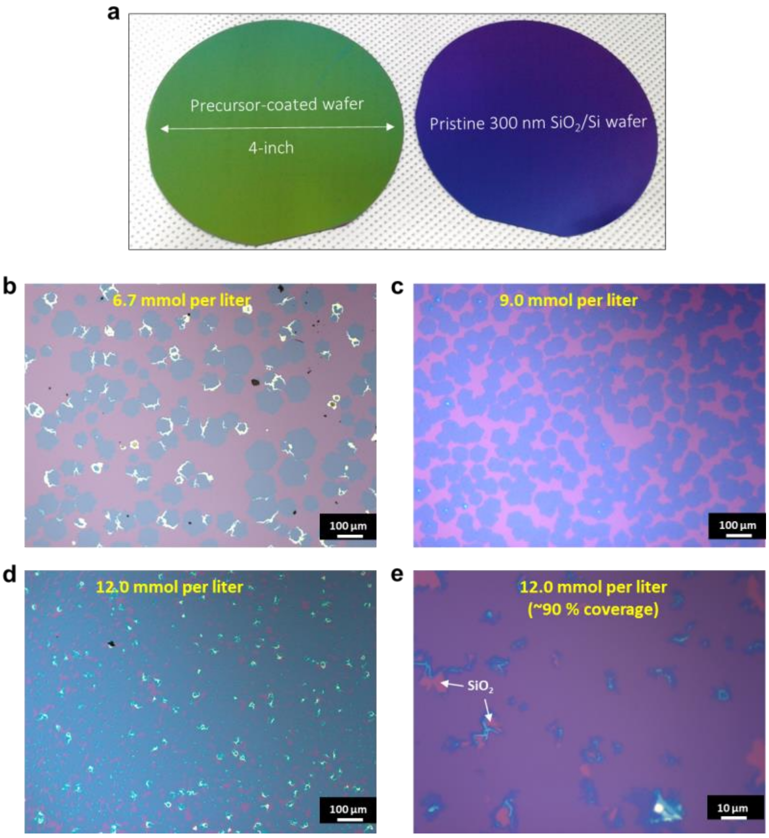


**Figure S1.** (a) Photograph image of the liquid precursor coated (left) and pristine 300 nm $SiO_2$/Si wafer (right). (b-e) Optical images of the CVD-grown $WSe_2$ with different concentrations of W-chemical in the liquid precursor. With increasing the mole concentration of W-chemical in the liquid precursor, the coverage of $WSe_2$ is higher and the $WSe_2$ film can be achieved with 90% coverage at 12.0 mmol per liter.

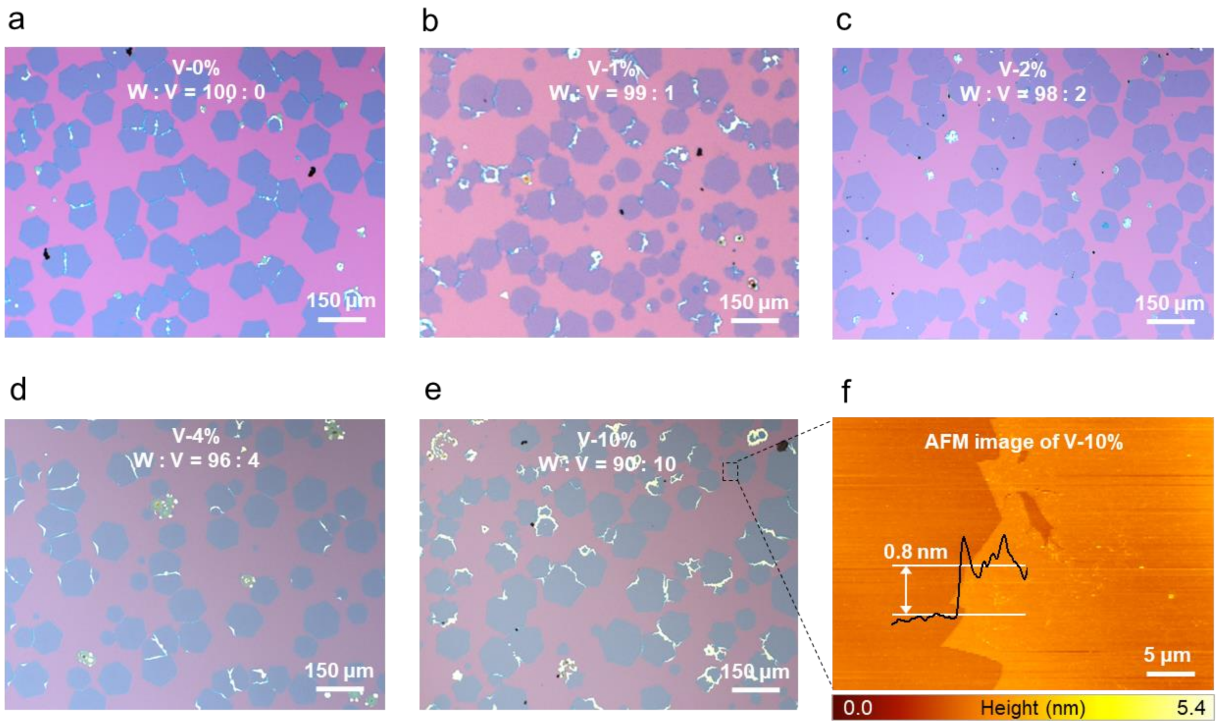


**Figure S2.** (a-e) Optical images of V-doped $WSe_2$ monolayer. The monolayer thickness of V-doped $WSe_2$ is maintained up to V-10% doping (nominal value of V-concentration), as confirmed by (f) AFM.

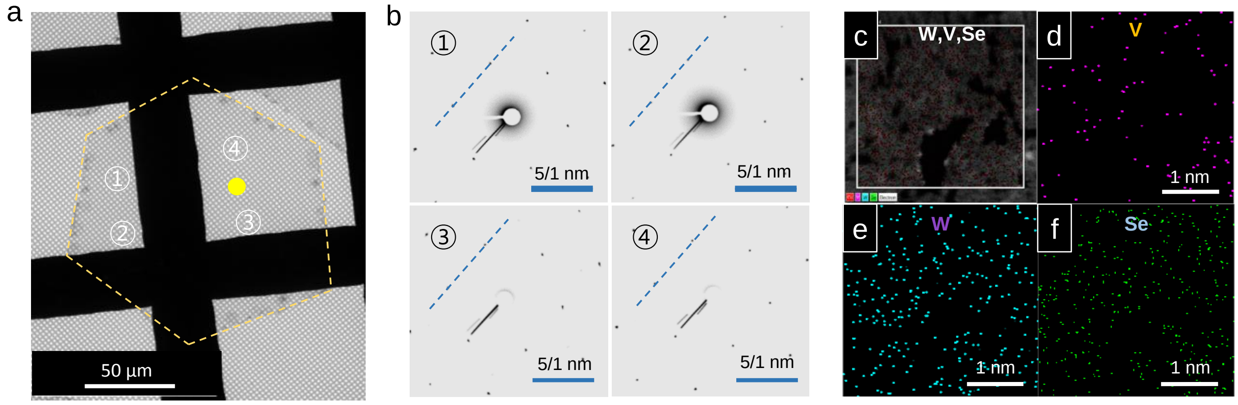


**Figure S3.** (a) Low magnification TEM observation and (b) diffraction patterns of V-10% doped $WSe_2$. The diffraction patterns with the same orientation indicate single crystalline V-doped $WSe_2$ flake. (c-f) EDS mapping images of yellow region in (a) for each element (V,W, and Se, respectively).

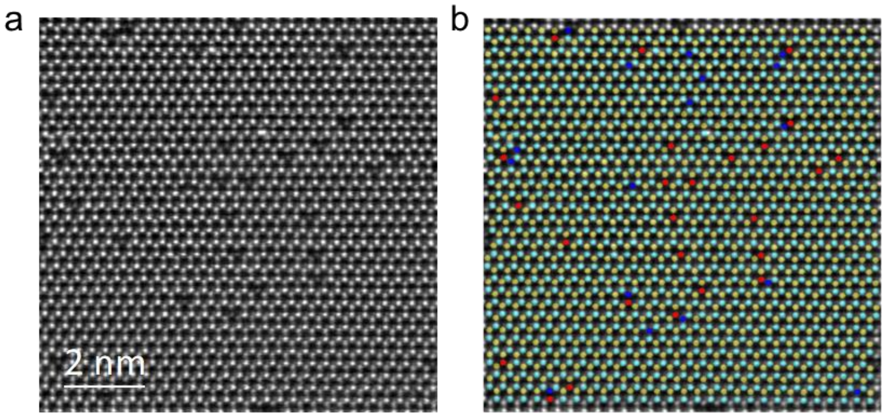


**Figure S4.** STEM images of the V-2% doped $WSe_2$ after (a) the Gaussian-blur filtering and (b) atomic mapping. W atom (cyan), V atom(red), 2Se atoms (yellow), and Se-vacancy (blue) are clearly distinguished. The extracted value of the defect density is 1.7 $\times$ $10^{13}$ $cm^{-2}$.

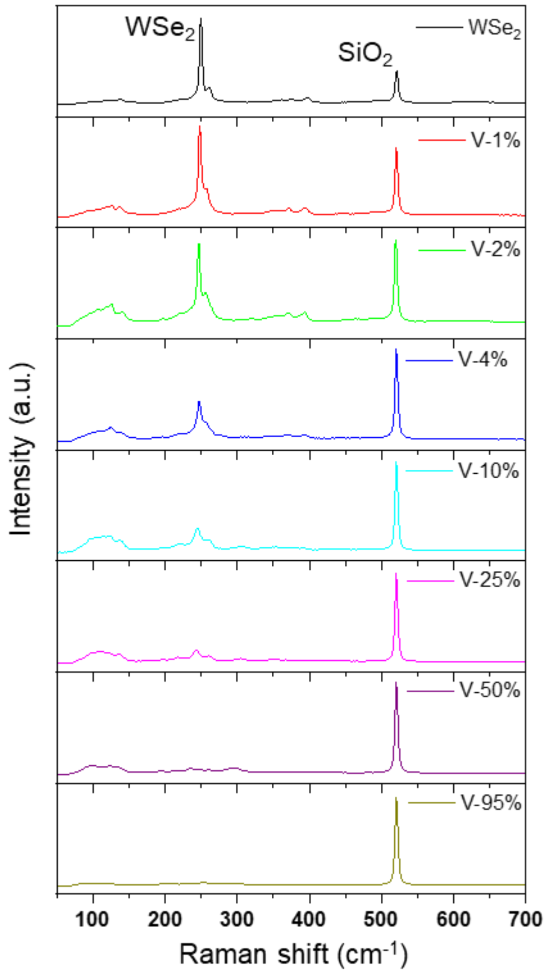


**Figure S5.** Raman spectra of V-doped $WSe_2$ with different V-concentrations.

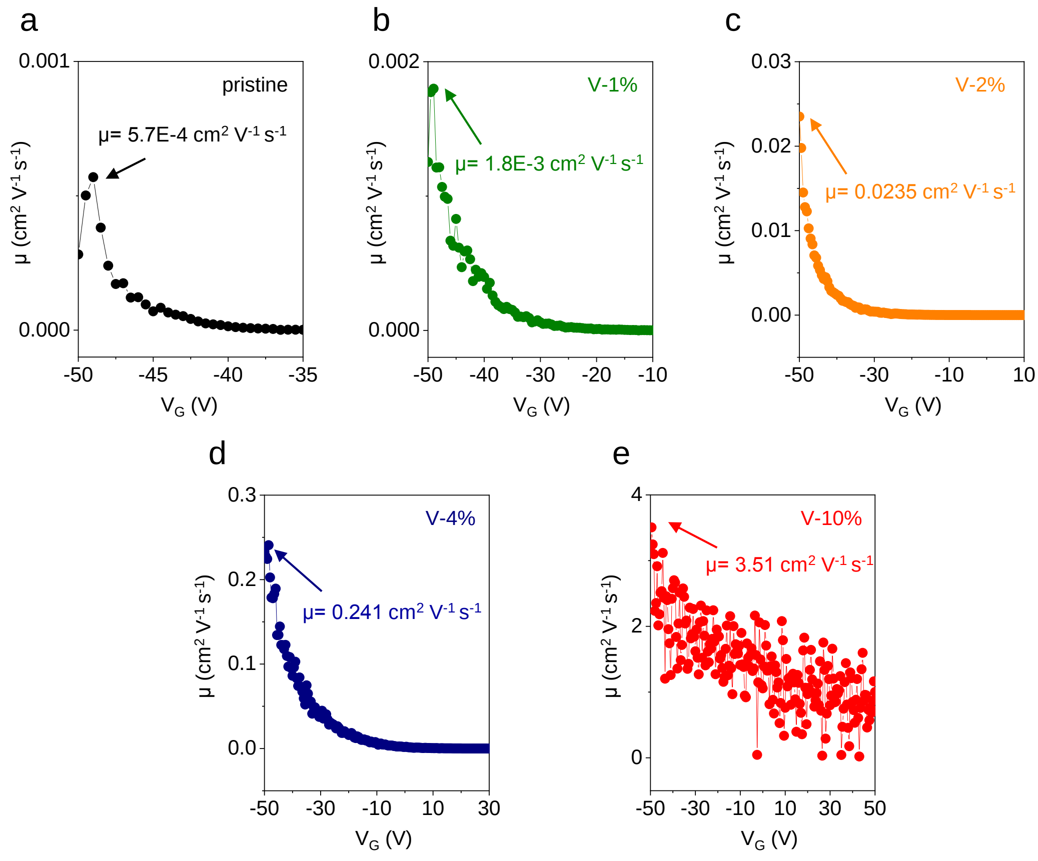


**Figure S6.** Field effect hole mobilities as a function of gate bias in (a-e) pristine $WSe_2$, 1%, 2%, 4%, and 10% V-doped $WSe_2$-FETs, respectively.

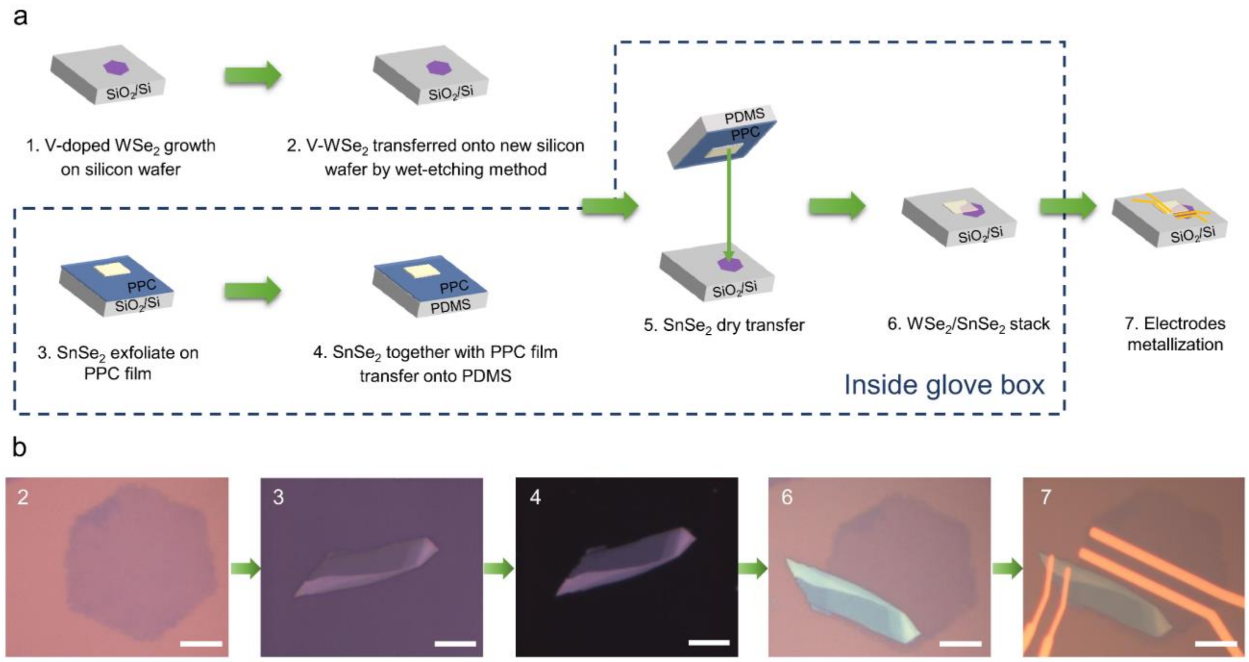


**Figure S7.** Fabrication process of V-doped $WSe_2/SnSe_2$ p-n diodes on the $SiO_2$ substrates. (a) Schematic illustration of the whole process. (b) Optical microscopy images with the numbers in the upper-left corner corresponding to each fabrication step in (a). The scale bar is 10 μm.

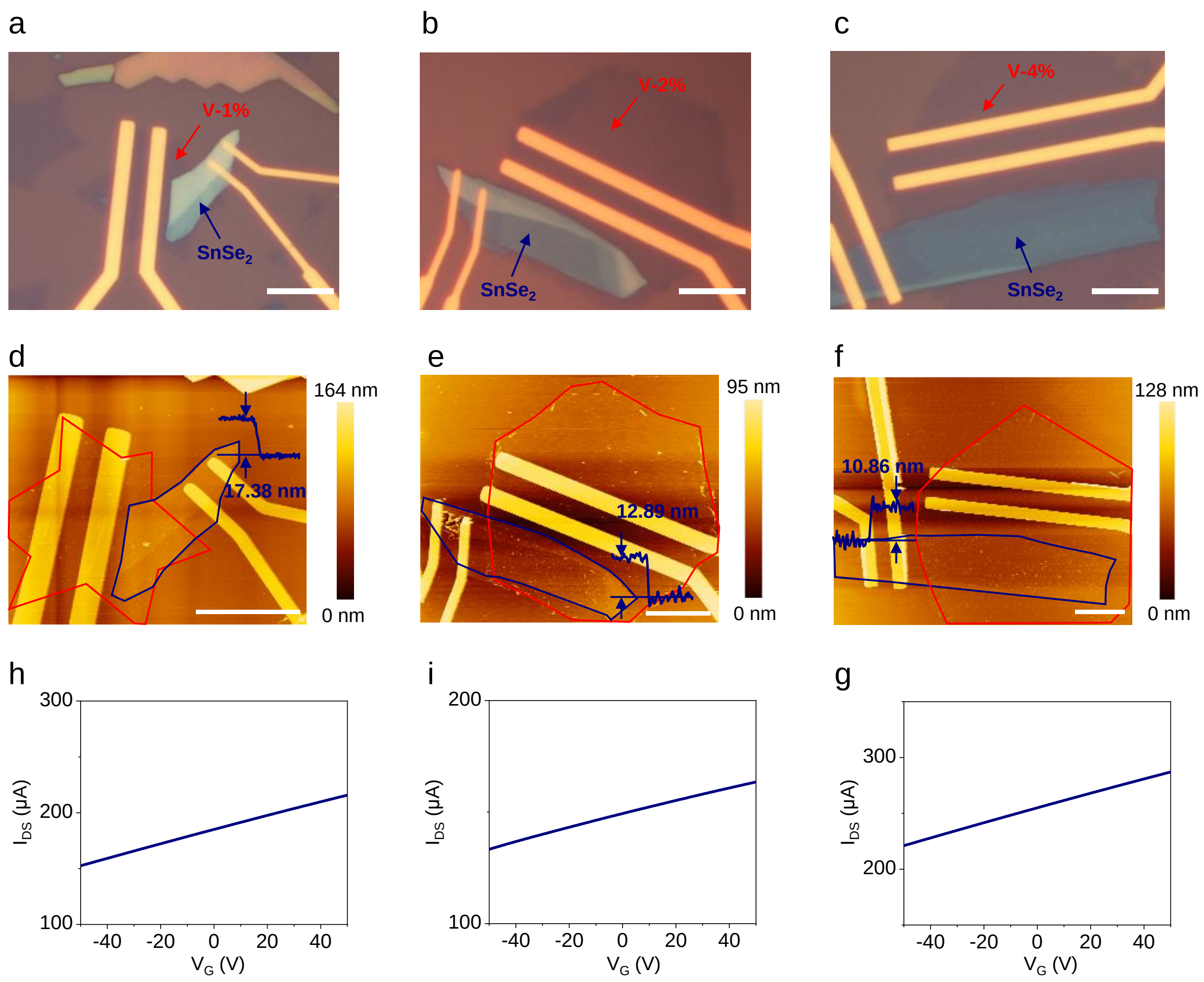


**Figure S8.** Characterization of $SnSe_2$ flakes as n-type materials. (a-c) OM images of V-1%, V-2%, and V-4% devices, respectively. The scale bar is 10 μm. (d-f) AFM mappings of each device, confirming the thickness of $SnSe_2$ flakes ranging from 11 to 17 nm. (h-g) Transfer curves of each individual $SnSe_2$-FET corresponding to the devices in (a-c), exhibiting identical degenerate *n*-type properties with the same current level. The drain bias is fixed at 0.5 V.

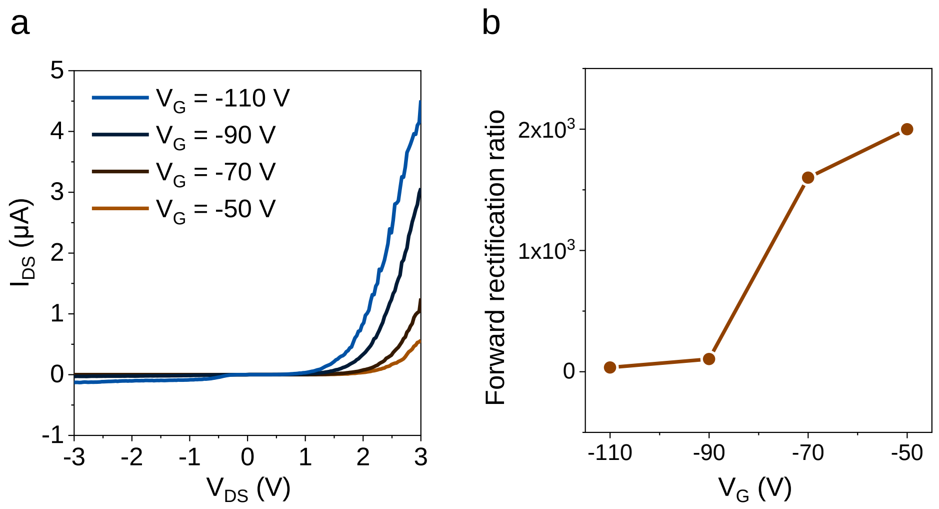


**Figure S9.** Electrical characteristics of pristine $WSe_2/SnSe_2$ diode. (a) $I_{DS}$-$V_{DS}$ output curves at various back-gate voltages (from -50 V to -110 V, $WSe_2$ in *p*-type on-state), showing the forward rectifying behavior. (b) Forward rectification ratio as the function of gate bias.

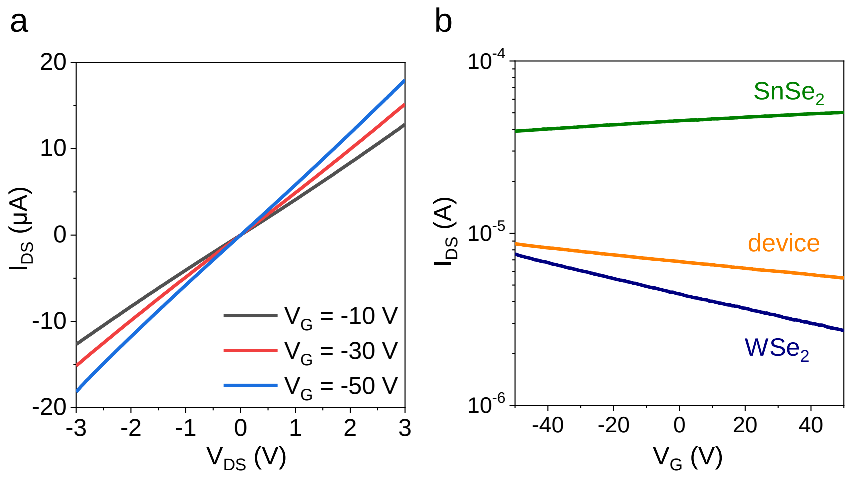


**Figure S10.** Electrical characteristics of V-10% $WSe_2$/$SnSe_2$ device. (a) $I_{DS}$-$V_{DS}$ output curves at various gate biases, showing ohmic resistance in both forward and negative drain bias region. (b) Transfer curves of V-$WSe_2$ FET, $SnSe_2$ FET, and device with weak gate tunability.

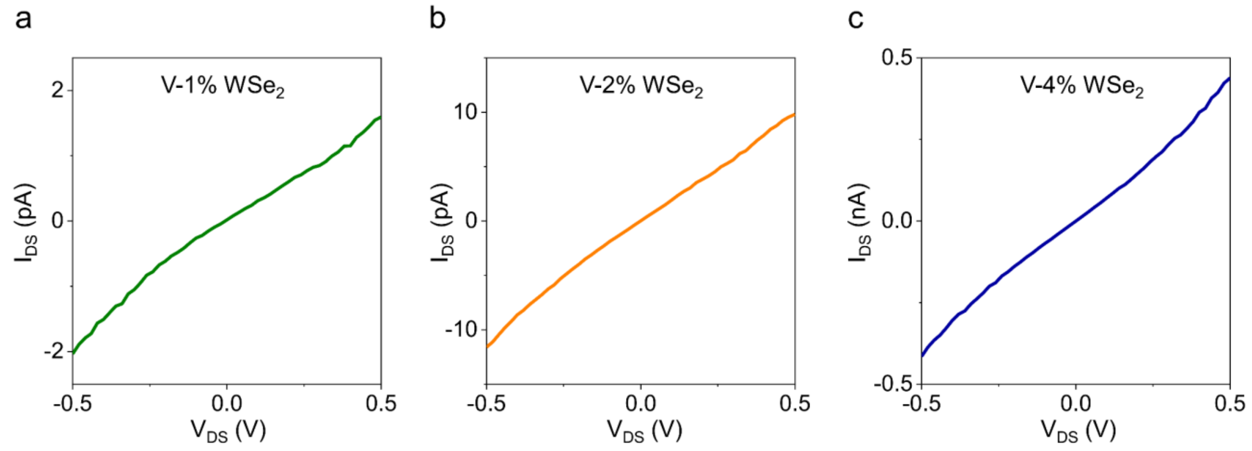


**Figure S11.** (a-c) Near-ohmic contact in the 1%, 2%, and 4% V-$WSe_2$/Pd junctions, respectively. All the $WSe_2$ flakes are gated at -20 V for hole carrier transport.

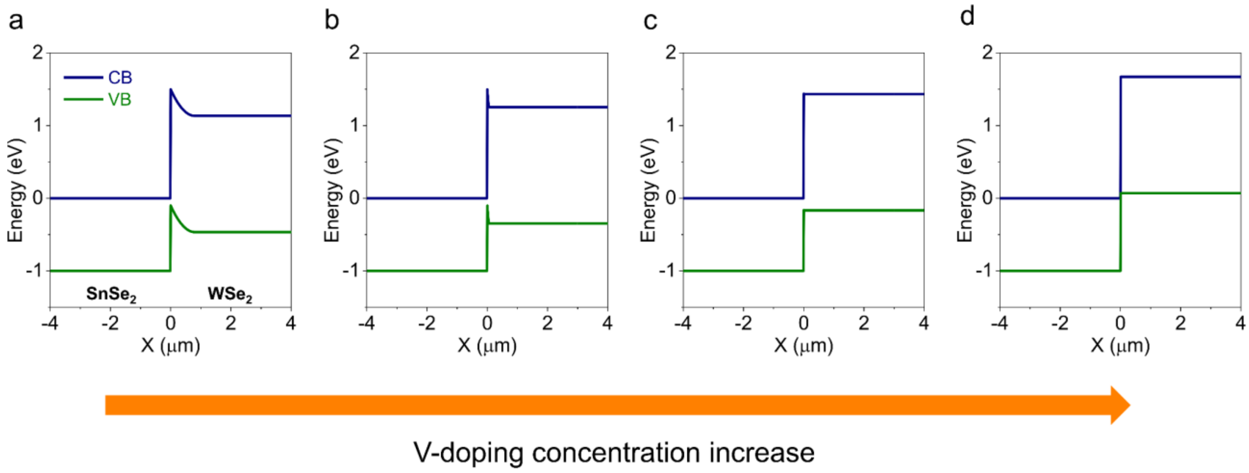


**Figure S12.** (a-d) Band alignment simulations using Poisson's equation by gradually increasing $N_a$ by $10^{14}$ cm$^{-3}$, $10^{17}$ cm$^{-3}$, $10^{19}$ cm$^{-3}$, and $10^{23}$ cm$^{-3}$, respectively.

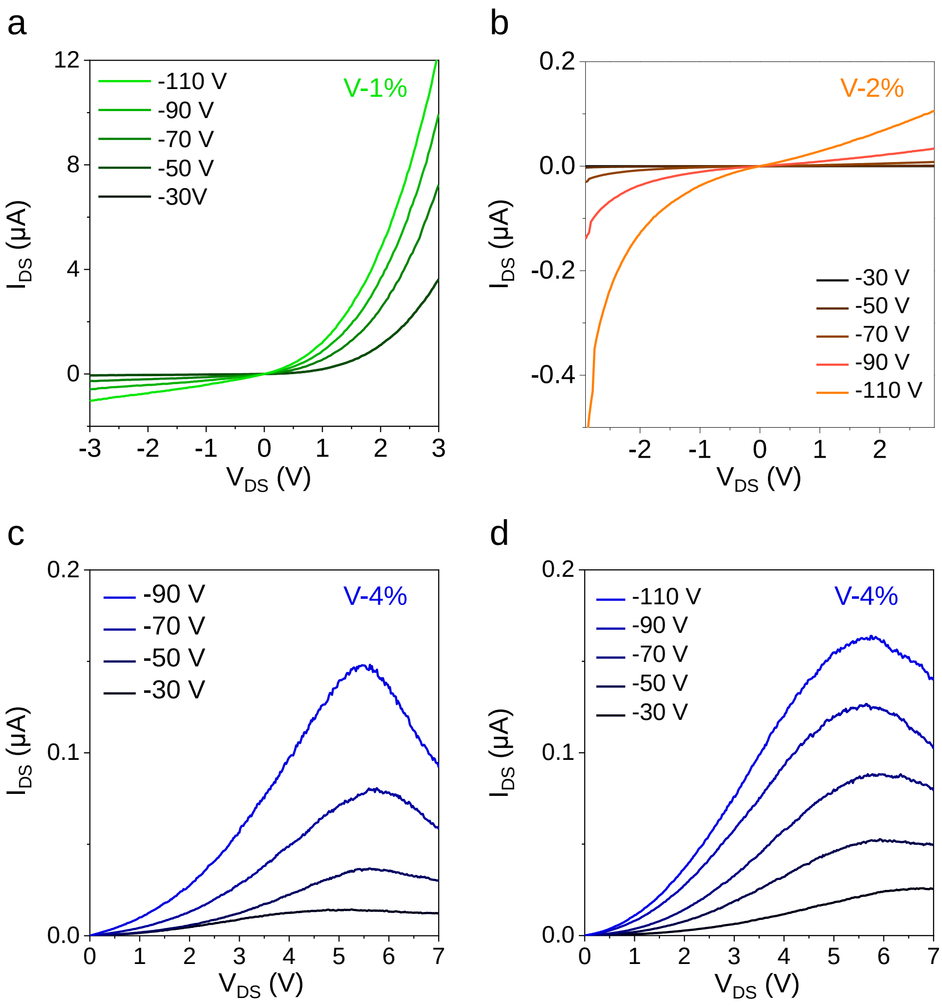


**Figure S13.** Reproducibility of (a) V-1% diode with the forward rectifying behavior, (b) V-2% diode with the backward rectifying behavior, and (c-d) V-4% devices with NDR tunneling at various gate biases.

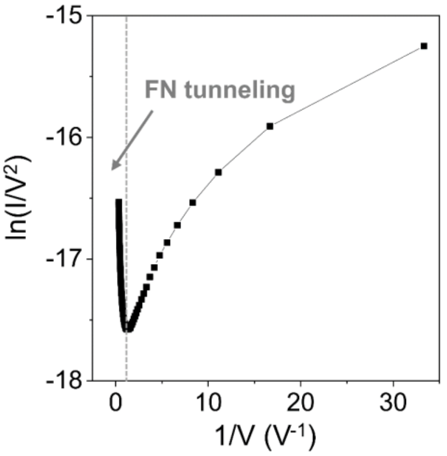


**Figure S14.** The ln($I$/$V^2$)−1/$V$ plot of $I_{DS}$-$V_{DS}$ curve in Figure 3d, confirming the carrier transport of FN tunneling or thermionic FN tunneling with a negative slope at a high drain bias (near zero).

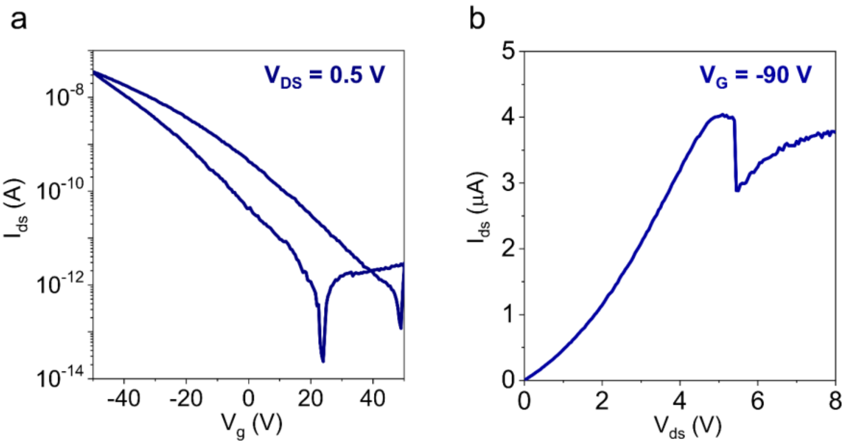


**Figure S15.** (a) Large hysteresis in V-4% $WSe_2$-FET. (b) Device with apparent $I_{DS}$ pick-up behavior.
